# The long lost denture: a rare case of an acquired, non-malignant tracheo-oesophageal fistula

**DOI:** 10.1186/s13019-024-03073-3

**Published:** 2024-11-04

**Authors:** Hannah Jesani, Aaron Hundle, Paul Nankivell, Maninder Kalkat

**Affiliations:** 1 Department of Thoracic Surgery, University Hospitals Birmingham NHS Foundation Trust, Birmingham, UK; 2 Department of Otolaryngology, University Hospitals Birmingham NHS Foundation Trust, Birmingham, UK; 3 University of Birmingham, Institute of Cancer and Genomic Sciences, Institute of Head and Neck Studies and Education (InHANSE), Birmingham, UK

**Keywords:** Tracheo-oesophageal fistula (TOF), Denture, Delayed presentation, Tracheal resection, Oesophageal diversion, Gastric pull-up, Multidisciplinary team

## Abstract

**Background:**

Ingested dental prosthesis are susceptible to impaction in the gastrointestinal tract due to their sharp edges, size and contour. Delays in presentation arise from the lack of clear history of ingestion and misdiagnosis occurs due to the radiolucency of denture material on plain radiography. An acquired, non-malignant tracheo-oesophageal fistula (TOF) may develop from a chronically impacted denture. Surgical management of a TOF secondary to denture is a challenging clinical problem that is rarely reported in the literature and no previous case reports have described the two-staged reconstruction approach that we present here.

**Case presentation:**

We report a case of a male in his early 60s who presented to an acute general hospital with symptoms ongoing for over one year of dysphagia, recurrent chest infections and weight loss. Barium swallow and computed tomography identified an ingested dental prosthesis (denture) that had caused a TOF. He was transferred to our specialist thoracic surgery unit where an attempt to remove the foreign body endoscopically was abandoned due to firm impaction and risk of further injury. The subsequent multi-disciplinary management of this complex case required a two-staged reconstruction approach. The first procedure involved extracting the foreign body, repairing the underlying defects with tracheal resection and anastomosis, and creating an oesophageal diversion with cervical oesophagostomy. The second procedure achieved continuity of the gastrointestinal tract with gastric pull-up and pharyngo-gastric anastomosis. Following rehabilitation, the patient was discharged on oral intake alongside percutaneous jejunostomy feeding.

**Conclusions:**

Early recognition and removal of impacted dental prosthesis is essential to prevent morbidity and mortality. Delayed diagnosis can lead to acquired TOF with associated consequences such as recurrent pulmonary infection, mediastinitis and nutritional deficit. Challenges we encountered, such as failed attempts at endoscopic retrieval and the difficult dissection of fibrotic tissue, were directly due to the delayed identification of the denture. We highlight the importance of holding a high index of clinical suspicion of foreign body ingestion in dental prosthesis wearers who present with recurrent chest infections and ongoing dysphagia. We also promote the need for a collaborative multi-disciplinary approach in the surgical management of complex cases.

## Background

Acquired tracheo-oesophageal fistula (TOF) is a rare pathology that most often arises from malignant disease in the adult population [1]. The aetiology of benign TOF is more varied and may include trauma, iatrogenic injury, foreign bodies, corrosive injury, oesophageal diverticula or local infection [2, 3]. Dental prostheses such as dentures constitute a sharp foreign body [4], with a size and contour that makes them susceptible to impaction in the gastrointestinal tract [5]. A national Dental Health Survey showed that dentures are worn by 20% of the United Kingdom population [6]. Due to an ageing population this figure will rise further on account of the higher prevalence of denture use in adults aged above 65 years [4]. The risk of complications from impacted dentures is most notably related to the duration of time since ingestion episode and the site of impaction [5, 7, 8]. The oesophagus is the most reported site [4] with complications including perforation and fistula formation [9]. Factors contributing to the delay in diagnosis include a lack of definite history from patients [10, 11] and the radiolucency of denture material [2, 5, 8]. TOF is a challenging clinical problem that carries significant long-term morbidity due to interference with nutrition and the pulmonary sequelae of ongoing tracheobronchial contamination [3, 12]. We report a case of a delayed presentation with an impacted denture causing a secondary, acquired, non-malignant TOF. We describe a multi-disciplinary team (MDT) approach to remove the denture foreign body, repair underlying defects and ultimately achieve continuity of the digestive tract. A two-staged reconstruction in this manner has not been previously reported for such an indication.

## Case presentation

**Fig. 1:**
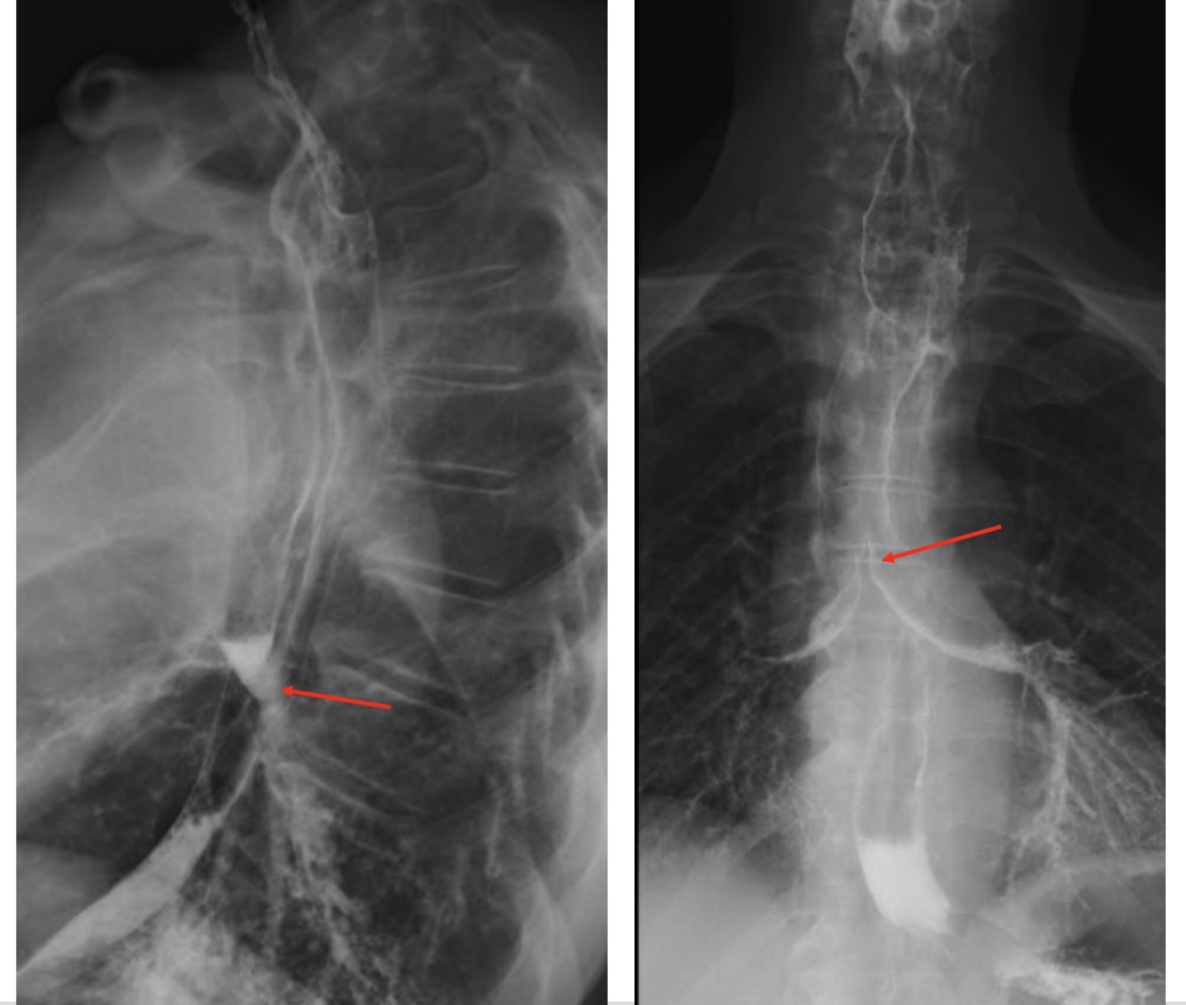
Pre-operative barium swallow fluoroscopy study. Shows contrast in trachea and oesophagus (red arrows) suggesting a fistulous communication

**Fig. 2:**
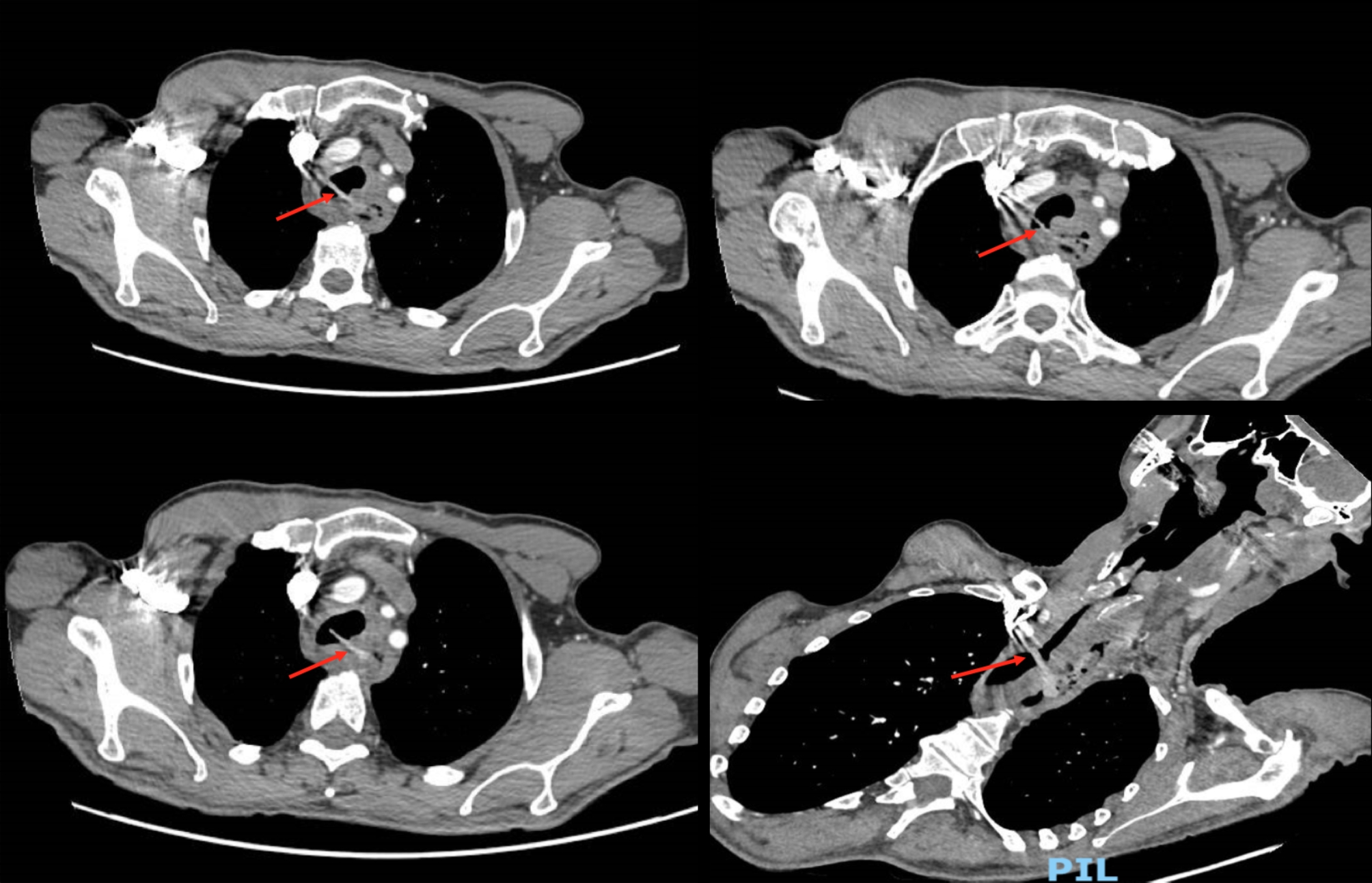
Axial slices of CT scan with contrast. Shows a linear hyperdense shadow extending from oesophagus to proximal trachea within a tracheo-oesophageal fistulous tract at the level of T2 vertebrae

A male in his early 60s attended the emergency department in an acute general hospital complaining of coughing and regurgitation after eating. He reported a history of dysphagia, initially to solids then liquids, weight loss and recurrent chest infections. These symptoms were ongoing for over a year, worsening in a two-week period prior to presentation. The patient did wear a dental prosthesis but did not recall an episode of swallowing a denture. Past medical history included epilepsy, well controlled with sodium valproate. The patient did not smoke, occasionally drank alcohol and was independent with activities of daily living. Barium swallow fluoroscopy reported an irregularity of the oesophageal tract, aspiration of contrast on swallowing and findings of an oesophageal mass [Fig. 1]. Computed tomography (CT) scan of neck, thorax, abdomen and pelvis with contrast reported oesophageal wall thickening suggesting a fistula between oesophagus and proximal trachea as well as bilateral ground glass shadowing secondary to infection [Fig. 2]. Notably there was no mention of a foreign body in the initial report. Oesophagogastroduodenoscopy (OGD) commented on difficult visualisation at 19 centimetres from incisor, showing a ‘large, stalked polyp’ like lesion at this high level with difficulty obtaining a tissue biopsy.

The patient was referred to our specialist thoracic surgery unit at a large tertiary centre. Review of imaging established the presence of a foreign body across the fistulous tract. Endoscopic retrieval was attempted using flexible and rigid bronchoscopy as well as oesophagoscopy. The procedure was abandoned after recognising that the foreign body was in fact a denture with sharp edges, and due to firm impaction, manipulation would cause bleeding and further injury to the trachea or oesophagus. Instead, the MDT decided to prepare the patient for a planned open surgical procedure to remove the denture and establish the integrity of the aerodigestive tract.

### First procedure

**Fig. 3:**
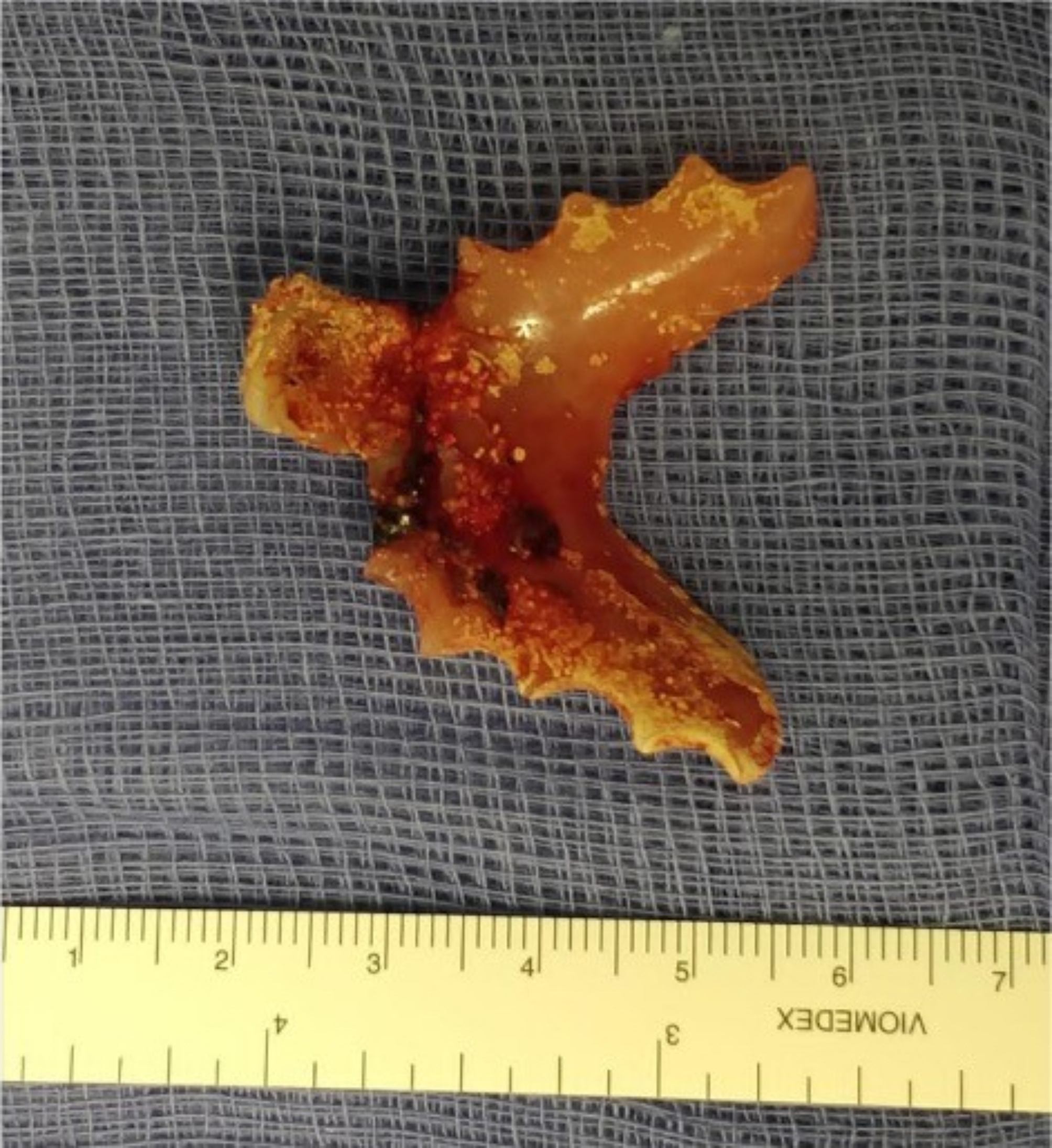
The extracted foreign body - a removable, partial denture

Access to the fistulous site was achieved via a right posterolateral thoracotomy through the fourth intercostal space. The mediastinum was inflamed and fibrotic due to persistent contamination, with the trachea densely adherent to the oesophagus. The fistula was reached via tracheotomy and denture foreign body extracted [Fig. 3]. The airway was maintained by using cross ventilation with a reinforced endotracheal tube positioned in the distal trachea. A tracheal resection was performed, removing 2 cm non-viable tracheal tissue, with an end-to-end anastomosis of healthy proximal and distal trachea. A pedicled anterior pericardial fat patch was laid over the anastomosis to reinforce the repair and promote healing. The oesophagus was divided in the chest with distal end stapled and the proximal part dissected up to the root of the neck. The thoracotomy was closed after placing a chest drain. The patient was turned supine and cervical oesophagus exposed in the left side of the neck. The dissection of the proximal oesophagus proved challenging due to dense fibrotic adhesions with trachea and surrounding structures. A cervical oesophagostomy was performed. A percutaneous endoscopic feeding gastrostomy (PEG) was placed to provide nutritional support, optimising the patient prior to further surgical intervention. This was deemed a suitable enteral feeding route in the interim, instead of a feeding jejunostomy which would have required refashioning during the secondary reconstruction.

### Complicated postoperative recovery

Following the procedure, the patient encountered several complications. Immediately after extubation the patient rapidly desaturated and flexible nasendoscopy revealed bilateral vocal cord palsy, presumably due to recurrent laryngeal nerve damage. He underwent emergency re-intubation and was admitted to the intensive care unit (ICU), where he struggled to wean from ventilation. Subsequently, a microlaryngoscopy and coblation of the posterior glottis was undertaken to improve airway obstruction but he ultimately required a surgical tracheostomy. A few weeks later, the patient had recurrent episodes of desaturation with thick creamy secretions in the airway. Bronchoscopy revealed thick white casts blocking the segmental bronchi, particularly on the right side. This was confirmed to be retrograde aspiration of the PEG feed through new fistulisation of the distal stapled oesophagus to lung parenchyma. This was controlled with laparoscopic stapling of the gastro-oesophageal junction. The patient was later stepped down to the ward after spending 62 days in the ICU. Another significant issue was cicatrisation and stenosis of the cervical oesophagus leading to failure to drain the saliva into stoma bag externally. This resulted in persistent aspiration of secretions through the dysfunctional larynx whenever the tracheostomy tube cuff was deflated.

### Second procedure

**Fig. 4:**
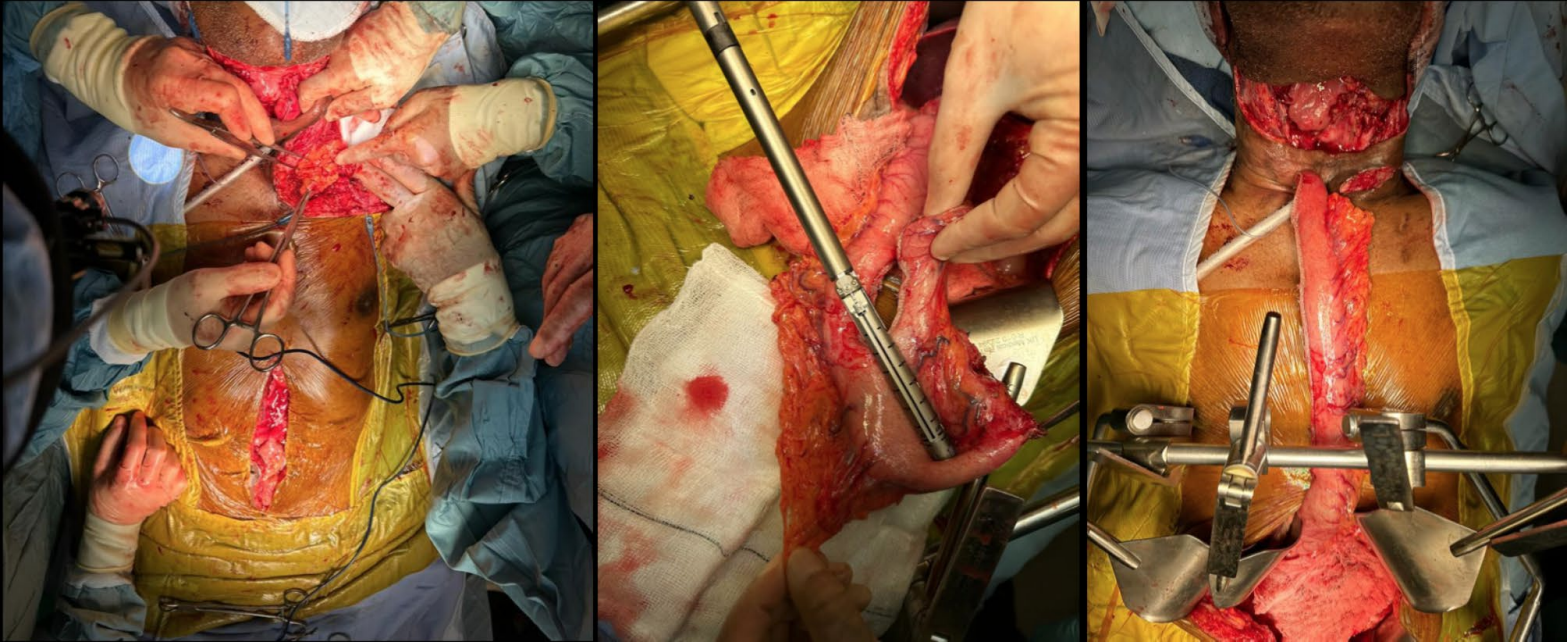
Creation of the gastric pull-up. Images show the mobilisation of stomach (left), formation of gastric tube (middle) and retrosternal passage of conduit (right)

**Fig. 5:**
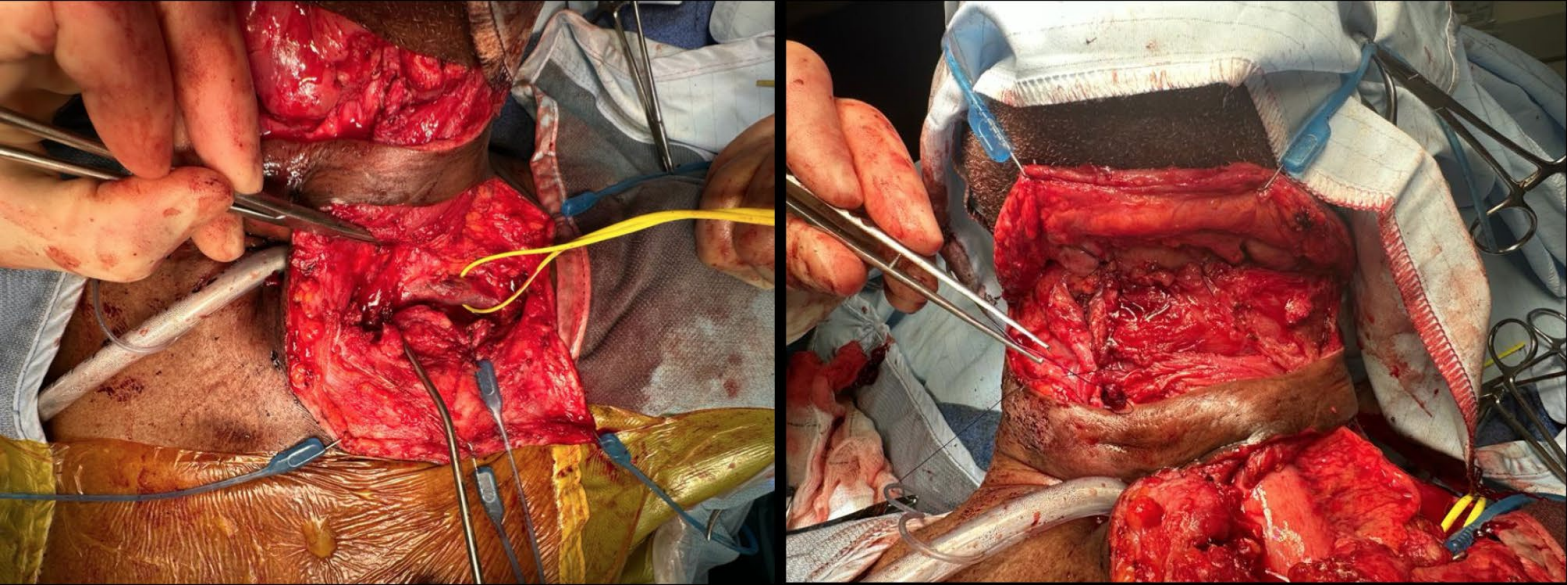
Restoring digestive tract continuity. Images show establishing left clavicular access (left) and the formation of pharyngo-gastric anastomosis (right)

The MDT recommended pharyngolarnyngectomy, permanent tracheostomy and restoration of the digestive tract. A U-shaped incision was made above the tracheostomy site, the pharynx was freed from prevertebral fascia and the larynx dissected en bloc. Sutures were placed into the third tracheal ring and pulled up to the laryngeal stoma thus creating a deep stoma. Through midline laparotomy, the stomach was fully mobilised with Kocherisation of the duodenum and clearance of adhesions posteriorly from the pancreas up to the gastroduodenal artery. A healthy gastric tube conduit was fashioned using staples. A tunnel was created with blunt dissection posterior to the sternum for the passage of the gastric conduit [Fig. 4]. A partial manubriectomy with resection of medial ends of the left first rib and clavicle was performed. This created adequate space for the conduit and pharyngo-gastric anastomosis without tension and pressure at the thoracic outlet [Figure 5]. Finally, a feeding jejunostomy was placed via a small enterotomy.

### Outcome

**Fig. 6:**
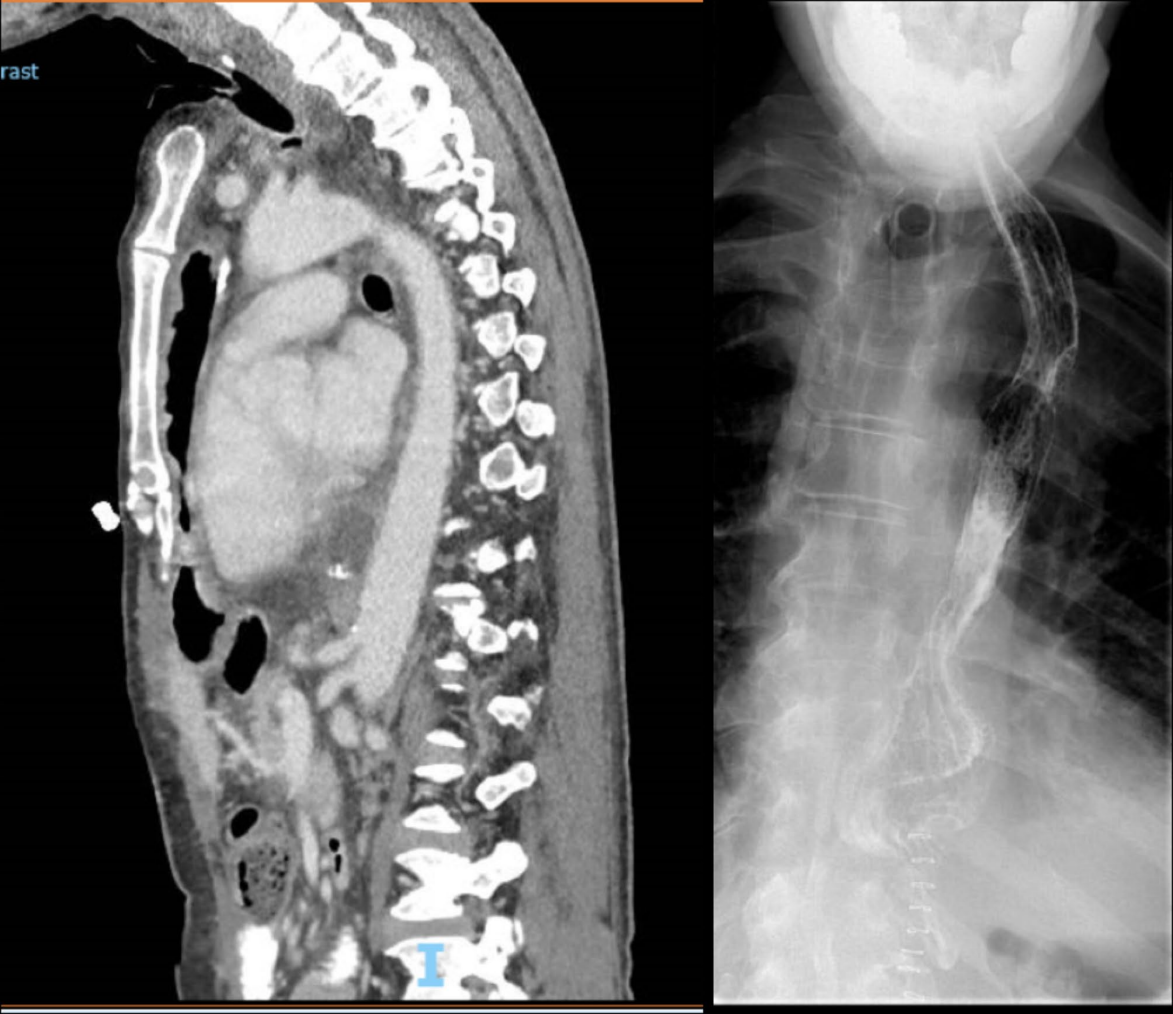
Post-operative radiological images. CT scan (left) showing retrosternal passage of reconstructed gastric tube conduit. Barium swallow (right) showing no leak from superior pharyngo-gastric anastomosis

Overall inpatient recovery and rehabilitation took place over a period of 8 months from the time of the first procedure. Inpatient support included the speech and language therapy, dietician, physiotherapy and counselling teams. Twelve days after the second stage procedure, a CT scan and barium contrast swallow identified no leaks from the pharyngo-gastric anastomosis [Fig. 6]. Oral intake was gradually built up alongside percutaneous jejunostomy feeding in time for discharge. Outpatient clinic review showed that the patient was eating and drinking a varied consistency, gaining weight and strength, vocalising with use of an electrolarynx device.

## Discussion and conclusions

Early recognition of an impacted foreign body in the aerodigestive tract and early diagnosis of related complications are important for the success of treatment approaches [8, 9, 13]. Prolonged impaction in oesophagus leads to a pathological process of oedema, mucosal infection and necrosis [7]. In our case, missed identification of the denture allowed it to erode into the airway and become lodged in a fistulous connection contributing to repeated aspiration, mediastinitis and failure to thrive. This led to the challenges we encountered such as failed endoscopic retrieval and the difficult dissection of fibrotic tissue, which contributed to iatrogenic recurrent laryngeal nerve injury during the first procedure. In anticipation of this difficult surgical field, we could have considered intraoperative nerve monitoring, which may have helped to preserve phonation.

Several factors influence the difficulty with identifying impacted dentures. In our case, the patient provided no clear account of swallowing a denture. Unnoticed ingestion is not uncommon and in a 10-year review of ingested dentures including 85 cases, 18% of patients were unaware of swallowing their denture, with 40% of this subgroup having cognitive impairment [4, 10]. We also know that patients with learning disabilities or mental health disorders may be unable to give an accurate history of the event [5, 7]. However, the patient did not have any documented risk factors for unnoticed denture ingestion other than a personal history of epilepsy. It is possible that the patient may have swallowed their denture during a seizure episode. We recognise the need to hold a high index of clinical suspicion for foreign body ingestion in dental prosthesis wearers who present with persistent dysphagia or recurrent chest infections. Popular acrylic dentures are made from radiolucent plastic material [7, 9] and unless they contain metal attachments it is not easy to directly visualise them on plain radiographs [2, 5, 7]. CT is the primary investigation of choice because it is more sensitive at detecting dentures [7] and may identify complications such as perforation, TOF, abscess formation or mediastinitis [2, 5]. 

Definitive surgical treatment is certainly required for an impacted foreign body with overt complications such as an acquired, non-malignant TOF [13]. Requirement for open surgery is more likely with a longer duration between ingestion event and diagnosis [4]. There are several approaches to surgical management, which can be broadly categorised into single stage procedures for exploration and repair of the TOF, with or without tracheal resection; or ‘exclusion’ procedures involving primary closure of the tracheal defect and oesophageal diversion, without a later second stage reconstruction [3, 14]. The most important influences on choice of approach are underlying aetiology, site of the fistula and state of the affected tissue [14]. Shen and colleagues reported on a case series of 56 operations in 35 patients at a single institution for non-malignant TOF, recommending the safety of single-stage repair of both airway and oesophageal defects with pedicled tissue flap interposition [3]. However, this series did not include any erosions from a foreign body or dental prosthesis. In our presented case, the defect in the trachea was large with inflamed edges, necessitating resection and anastomosis of healthy tissue.

We could only find one existing case report similar to our own that described a late presentation of TOF secondary to swallowed denture [2]. Samarasam and colleagues reported a challenging surgical field complicated by ‘peri-oesophageal sepsis with local abscess formation’ which informed their operative decision to perform a subtotal oesophagectomy and cervical oesophago-gastric anastomosis. The perioperative period in our own case was associated with several adverse outcomes including bilateral vocal cord palsy, dysfunctional oeosphageal stoma and pulmonary-thoracic oesophageal remnant fistula, managed appropriately. This high morbidity was expected since complication rates are reported as high as 54.3% [3]. The most common specific complications include oesophageal leak (11.4%), recurrent TOF (8.6%), tracheal dehiscence (5.7%), vocal cord paralysis (5.7%) and mortality (5.7%) [3]. It is important to note that reconstruction techniques of the pharyngo-oesophageal junction have only been robustly analysed in contexts that differ from our report, such as cancer resection [15, 16] or corrosive oesophageal strictures [17]. 

Finally, we emphasise the importance of a collaborative MDT approach in the management of complications arising from denture ingestion and impaction. Successful repair of the TOF and restoration of digestive tract continuity required advanced surgical expertise available at our centre as well as diligent preparation and postoperative rehabilitation. The patient was subject to repeated physiological insults, permanent loss of phonation and negative psychological impact from his procedures and related complications. Regular input from speech and language therapy, dietician, physiotherapy and counselling teams was crucial for overcoming these challenges and reaching a condition that was safe for discharge.

In summary, we have reported on a complex case of an acquired, non-malignant TOF secondary to chronically impacted denture which is rarely described in the literature. It highlights the challenges associated with delayed recognition of an ingested dental prosthesis. We promote the importance of a broad MDT to optimally manage all aspects of work-up, surgical intervention and recovery. A robust restoration of the integrity of the airway takes precedence and may require oesophageal diversion to protect the repair site. We demonstrated a satisfactory outcome using this approach that included primary tracheal resection, tracheal anastomosis and temporary oesophageal diversion with cervical oesophagostomy, followed by a secondary reconstruction of digestive tract continuity with gastric-pull up.

## Data Availability

No datasets were generated or analysed during the current study.

## Abbreviations

CT: Computed tomography
ICU: Intensive care unit
MDT: Multi-disciplinary team
OGD: Oesophagogastroduodenoscopy
PEG: Percutaneous endoscopic gastrostomy
TOF: Tracheo-oesophageal fistula
